# A longitudinal qualitative study on the care needs journey map of primary caregivers of patients undergoing total knee arthroplasty

**DOI:** 10.3389/fresc.2025.1623623

**Published:** 2025-10-23

**Authors:** Jingru Hu, Guanglei Dong, Dongfang Liu, Dongyang Wang

**Affiliations:** ^1^ Department of Nursing, The Third People’s Hospital of Henan Province, Zhengzhou, China; ^2^ School of Nursing, North Henan Medical University, Xinxiang, China; ^3^ Department of Orthopedics, The Third People’s Hospital of Henan Province, Zhengzhou, China

**Keywords:** total knee arthroplasty (TKA), primary caregivers, longitudinal, qualitative research, caregiver burden

## Abstract

**Background:**

Total knee arthroplasty (TKA) is a common and effective treatment for advanced knee osteoarthritis, yet it imposes significant demands on primary caregivers throughout the perioperative and rehabilitation phases. Understanding caregivers’ evolving needs is critical for optimizing patient outcomes and sustaining caregiver well-being. This study aimed to explore the dynamic care experiences and changing needs of primary caregivers of TKA patients across three distinct phases: the diagnosis period, discharge transition, and rehabilitation phase, and to construct a comprehensive journey map of caregiving needs.

**Methods:**

A longitudinal qualitative study was conducted using purposive sampling. Sixteen primary caregivers of patients undergoing unilateral TKA were recruited from a tertiary hospital in Henan, China. Semi-structured interviews were conducted at three time points: preoperative (T1), pre-discharge (T2), and one month post-discharge (T3), resulting in 43 interviews. Data were analyzed using content analysis and synchronized temporal mapping to identify themes and subthemes along the caregiving timeline.

**Results:**

Four major themes and 27 subthemes were identified: care tasks, emotional experiences, caregiving barriers, and support systems. Caregivers’ responsibilities evolved from pre-surgical information gathering to intensive post-surgical care and long-term rehabilitation support. Emotional burdens shifted from anxiety and helplessness to fatigue and psychological strain. Major barriers included knowledge deficits, skill limitations, and inadequate systemic support. The caregiver support network transitioned from hospital-based to community and family-based systems over time. A visual journey map was developed to represent these findings.

**Conclusion:**

Primary caregivers of TKA patients face complex and changing needs across different stages of care. A caregiver-centered, multidisciplinary, and phase-specific support framework is essential to improve the quality of postoperative care and reduce caregiver burden.

## 1 Introduction

Knee osteoarthritis (KOA) is among the most common degenerative joint diseases in middle-aged and older adults, characterized by progressive cartilage loss and osteophyte formation that lead to persistent pain, functional limitations, and poorer quality of life (1). In China and globally, demographic aging, obesity, and lifestyle-related risks have contributed to steady rise in KOA burden, with older women disproportionately affected (2, 3). These trajectories not only reflect disease prevalence but also foreshadow escalating care needs at the household level.

For patients with advanced KOA, conservative therapies are frequently inadequate, and total knee arthroplasty (TKA) is widely regarded as the standard intervention to relieve pain and restore mobility (4). The procedure's diffusion has been rapid—millions of TKAs are performed worldwide annually, and China has seen double-digit growth since 2010 with continued expansion expected (5). While these trends underscore TKA's clinical value, they also magnify the volume and complexity of postoperative support required after discharge, when patients transition to home-based rehabilitation and rely heavily on informal care (6).

Primary caregivers—most often spouses or adult children—sustain recovery across multiple stages, from preoperative preparation and inpatient rehabilitation to transitional discharge and ongoing home care (7). Their responsibilities span assistance with activities of daily living, symptom monitoring, complication surveillance, facilitation of prescribed exercises, and provision of emotional support. Yet caregivers’ own needs—psychological coping, actionable information, and access to structured resources—are frequently under-recognized in routine care pathways (8).

A substantial body of work links caregiver burden and well-being with patient outcomes such as adherence, functional recovery, and readmission risk (9). However, much of this literature is cross-sectional, providing static snapshots that obscure how caregiving demands, stressors, and coping strategies evolve as patients move through distinct recovery phases. Limited longitudinal evidence illustrates marked temporal fluctuations—e.g., heightened strain in the first month and persistent information gaps thereafter—but the timing, intensity, and persistence of these patterns remain insufficiently resolved (10). Methodologically, heterogeneity in burden measures, follow-up intervals, and contextual factors further constrains synthesis, leaving uncertainty about when and how to intervene most effectively.

The “caregiving journey” framework addresses these limitations by foregrounding the staged, dynamic nature of informal care: responsibilities shift, emotions ebb and flow, and support needs reconfigure across perioperative and rehabilitation milestones (11). Building on this lens, journey mapping can make visible the “pressure points” where unmet needs cluster, thereby guiding the design and timing of targeted interventions and education (12). Nevertheless, systematic longitudinal applications of this approach in TKA remain scarce—particularly in China, where family-based caregiving is central—leaving gaps in culturally and system-specific guidance.

Accordingly, this study employs a longitudinal qualitative design to capture the evolving experience of primary caregivers for TKA patients (13). Repeated, in-depth interviews across preoperative, early postoperative, and home-rehabilitation stages allow us to trace temporal patterns in needs, stressors, and adaptive strategies beyond what cross-sectional snapshots can reveal. Guided by the gaps identified above, the objectives of this study are twofold: (1) to explore and interpret how caregivers’ needs, challenges, and coping processes evolve across the perioperative and rehabilitation stages of TKA; and (2) to construct an in-depth understanding of the critical moments where informational, practical, and psychosocial support needs become most salient, thereby providing a nuanced foundation for context-sensitive, stage-specific interventions. By prioritizing caregivers’ narratives and experiences, this study aims to generate empirically grounded insights for nursing practice, caregiver education, and health service planning.

## 2 Methods

### 2.1 Study design

This study adopted a longitudinal qualitative design grounded in the interpretivist paradigm, using the journey mapping method combined with conventional content analysis (14). This approach was selected to capture caregivers’ evolving experiences and to interpret the contextual meanings of caregiving practices over time. The design was further informed by SRQR (Standards for Reporting Qualitative Research) and COREQ (Consolidated Criteria for Reporting Qualitative Research) guidelines, to enhance transparency and methodological rigor (15).

### 2.2 Participants and sampling

Participants were the primary caregivers of patients who underwent TKA at Henan Provincial Third People's Hospital. A purposive sampling method, guided by the principle of maximum variation, was used to ensure diversity in age, gender, educational background, and socio-economic status. Inclusion criteria for patients were: (1) first-time recipients of unilateral TKA; (2) a Tampa Scale of Kinesiophobia (TSK) score >37, indicating moderate to severe kinesiophobia; (3) clear consciousness, normal cognitive and hearing function, and the ability to communicate verbally or in writing; (4) provision of informed consent and willingness to participate in longitudinal follow-up. Exclusion criteria included: (1) severe dysfunction of vital organs such as the heart, liver, lungs, or kidneys; (2) diagnosed anxiety, depression, or current use of psychiatric medications; (3) mental illness or cognitive impairments impairing communication; (4) simultaneous participation in other similar studies.

Inclusion criteria for caregivers were: (1) the primary caregiver providing ≥4 h of daily care; (2) unpaid caregiving role; (3) adequate communication and comprehension ability. Exclusion criteria included: (1) caregivers with a professional healthcare background; (2) patients’ death or caregiver withdrawal during the study. Sample size was determined according to the principle of data saturation, defined as the point at which no new codes or themes emerged during successive interviews (16). Saturation was reached after 16 caregivers were recruited, resulting in 43 interviews across three time points. The mean age of caregivers was 47.3 years (SD = 8.6). Ethical approval was granted by the Ethics Committee of Henan Provincial Third People's Hospital (Approval No. 2025-126).

### 2.3 Interview timing and guide

To reduce recall bias and ensure the authenticity of data, interviews were conducted at three key time points identified through literature review and clinical practice, and aligned with the structure of the caregiver journey map:

T1 (Preoperative phase): Within the first 2 days of hospitalization
T2 (Discharge transition phase): One day before discharge
T3 (Rehabilitation phase): One month post-discharge

Although these three time points may seem limited, they were chosen to balance participant burden with feasibility, and to capture phases shown by prior research to involve the most significant caregiving challenges. The relatively short interview duration (15–20 min) reflected participants’ time constraints and fatigue during hospital stays, but repeated interviews allowed for cumulative depth.

A semi-structured interview guide was used consistently, refined iteratively during data collection to accommodate emerging themes. Sample questions included: “What is your biggest current difficulty as a caregiver?” and “How has this experience affected your own life and health?” A pilot interview with two caregivers was conducted to refine question wording and flow.

### 2.4 Data collection

All interviews were conducted by two researchers trained in qualitative methods. One served as interviewer and reflexive facilitator, while the second took observational notes on non-verbal cues. The researchers positioned themselves as clinical nurses with prior experience in perioperative care, which facilitated rapport but also necessitated reflexive journaling to manage potential bias.

T1 and T2 interviews were face-to-face in a private hospital room, while T3 interviews were conducted in person or by telephone, depending on caregiver preference. All interviews were audio-recorded and transcribed verbatim within 24 h. Participants were anonymized using codes (N1–N16).

### 2.5 Data analysis

All interviews were transcribed verbatim within 24 h and independently verified by two researchers. Data were analyzed using conventional content analysis (17), supported by NVivo 12 software for coding and data management. The analysis proceeded iteratively: transcripts were read repeatedly for immersion, and open coding was conducted to generate initial codes. Codes with similar meanings were then grouped into subthemes and further synthesized into broader themes. These themes were subsequently mapped onto a temporal framework corresponding to the caregiving journey (T1–T3), enabling both synchronic and longitudinal interpretation. To ensure credibility, coding decisions and theme development were discussed in regular peer debriefing sessions, and an audit trail was maintained throughout. The interview guide was refined iteratively to incorporate emerging insights, and data collection continued until thematic saturation, defined as the absence of new codes in successive interviews, was achieved.

### 2.6 Rigor and quality control

To ensure methodological rigor, multiple strategies were implemented throughout the study. First, trust was established between researchers and participants through long-term engagement in inpatient nursing care, fostering an environment conducive to open and honest dialogue. Second, reflexive journaling was used by all researchers to document potential biases, emotional reactions, and decision-making during data collection and analysis. This practice helped minimize the influence of subjectivity and maintained analytical transparency. Third, member checking was employed by revisiting selected participants to verify the accuracy of thematic interpretations and confirm that the findings reflected their lived experiences. Lastly, to ensure technical integrity, dual-device recording was used during telephone interviews—one for communication and the other for audio capture—to prevent data loss and maintain recording quality. These quality assurance measures collectively contributed to the credibility, dependability, and confirmability of the study findings.

## 3 Results

This longitudinal qualitative study traced caregivers’ dynamic experiences across the preoperative (diagnosis), discharge transition, and rehabilitation phases (Table 1). The thematic analysis identified caregivers’ experiences and captured both barriers and facilitators (Table 2). The analysis yielded four main themes—care tasks, emotional experiences, caregiving barriers, and support systems—and 27 subthemes, which were integrated into a comprehensive caregiving journey map. This map visually demonstrated how caregivers’ roles evolved from performing basic organizational tasks to assuming a more complex “quasi-professional” function, accompanied by fluctuating emotions and shifting support needs.

### 3.1 Participant characteristics

A total of 16 dyads of TKA patients and their primary caregivers participated in this study. Caregivers ranged in age from 29 to 63 years, with a mean age of 47.3 years (SD = 8.6). The majority were female (68.8%), and most were either spouses (43.8%) or adult children (31.3%), with smaller proportions of in-laws (12.5%), siblings (12.5%), and parents of patients (6.3%). Educational attainment varied considerably, from illiteracy (12.5%) to postgraduate training (6.3%), though most caregivers had completed at least junior middle school (62.5%). Marital status was predominantly married (81.3%), with a minority unmarried (12.5%) or divorced (6.3%). Caregivers occupations were grouped into broader categories, including agriculture, healthcare and education, public service, technical or professional work, service and manual labor, and unemployed/retired, reflecting broad socio-economic heterogeneity.

Patients ranged in age from 52 to 74 years, with a mean age of 63.1 years (SD = 5.9), and an equal distribution of men and women. Educational attainment was generally lower than that of caregivers: nearly half (43.8%) had only primary schooling or were illiterate, while a minority reached junior middle school (37.5%) or higher education (18.7%). The majority were married (87.5%), and most were retiredd, agricultures, or manual workers. The primary causes of injury were falls from heights (31.3%), traffic accidents (25.0%), and slips (18.7%), with disease durations concentrated between one and three months (68.8%). Injury severity was most often classified as Grade B (31.3%) or Grade D (25.0%), while the remainder were Grade F as shown in Table 3.

**Table 1:** Map of the care needs journey of the primary carer of a TKA patient over three periods.

Themes and Subthemes	Disease diagnosis period (1–3 days)	Transitional period from hospitalization (7–14 days)	Recovery period (months to years)
Caregiver tasks	Handle admission procedures, accompany the examination, understand the surgical process and notify other family members	Prepare the family environment, master basic nursing skills and displacement skills, and prepare for discharge	Long-term support for patient rehabilitation training, management of common complications (such as pressure sores, thrombosis, etc.) and emotional support
Emotional responses	Shock, anxiety, helplessness; tension about the risks of surgery and the role change in care	Concerns about the effectiveness of rehabilitation, care fatigue, lack of confidence, and possible sleep disorders	Hope coexists with stress, and anxiety and frustration are felt about the slow, repeated recovery and long-term care
Barriers to caregiving	There is a lack of knowledge about TKA surgery and nursing process, and communication barriers are significant	They cannot master the key points of rehabilitation care and lack social support and rehabilitation guidance	The care task is long-term, the economic pressure is great, and the family responsibility conflict is obvious
Coordination with healthcare staff and departments	Orthopedics, anesthesiology, radiology, outpatient nurses, admission coordinators, family members	Nursing staff, physicians, rehabilitation therapists, medical social workers, family members and other caregivers	Health care workers in rehabilitation institutions, psychological counselors, community workers, volunteers, family support networks

**Table 2:** Themes of care tasks in three periods for the primary caregivers of TKA patients.

Period	Theme	Sub-themes	Encoding	Representative statements
Preoperative	Information preparation	Lack of medical knowledge	There is insufficient knowledge of preoperative procedures and anesthesia for TKA	N1: “The doctor said I had to have surgery. I didn't understand it very well, and I didn't know what anesthesia or joint replacement was.”
	Coping	Anxiety and worry	There is an underestimation of the risks of surgery and the pressures of care	N3: “I heard that the operation is risky, and I'm afraid he will hurt, so I'm always worried.”
	Hospital affairs	The process is difficult	Not familiar with hospital procedures and the workings of the health system	N5: “When I first entered the big hospital, I didn't know anything about the process. I had to run around to pay and get the bill.”
Hospital stay	Care participation	Assist with daily care	The patient is completely dependent on life in the early postoperative period, and the caregiver needs to be involved throughout	N7: “He can't move his legs. I have to help him put on his pants, eat and go to the bathroom.”
	Communication and coordination	Communication between nurses and patients is difficult	Information asymmetry makes it difficult for caregivers to obtain clear rehabilitation guidance	N11: “The doctor said it was too fast, I couldn't understand it, and I was afraid of asking too many questions and bothering him.”
	Emotional stress	Sleep and fatigue	Long-term care and mental stress lead to physical exhaustion	N9: “I can't sleep well at the hospital and I'm worried about his condition. I'm very tired.”
Post-hospitalization	Family care	Environmental adaptation is difficult	The home space and facilities are not suitable for postoperative rehabilitation needs	N2: “The stairs at home are too steep for him to get downstairs. There are no handrails in the bathroom.”
	Skill requirements	Lack of rehabilitation guidance	Lack of guidance on postoperative functional training methods, frequency and precautions	N13: “The doctor said to exercise, but he didn't teach me how to do it or how long to do it.”
	Role conflict	Time allocation pressure	Multiple role (work, family) conflicts increase the pressure of care	N6: “I have to go to work and take care of him. I don't have enough time.”

**Table 3:** General information of TKA patients and their caregivers (*n* = 16).

Number	Relationship with patients	Sex	Age (years)	Educational status	Marital status	Occupation	Patient gender	Age of patient (years)	Educational level of patients	Marital status of the patient	Patient occupation	Disease duration (months)	Damage grading	T1	T2	T3
N1	Spouse	Woman	50	Junior college	Married	Healthcare/education	Man	65	Senior middle school	Married	Unemployed/other	2	3	F	F	F
N2	Sons and daughters	Man	32	Undergraduate course	Unmarried	Technical/professional	Man	70	Junior middle school	Married	Retired	1	3	F	F	P
N3	Son's wife	Woman	38	Undergraduate course	Married	Finance	Man	72	Primary school	Married	Agriculture	3	3	F	F	F
N4	Spouse	Man	60	Special school	Married	Service/manual labor	Woman	62	Senior middle school	Married	Retired	2	3	F	F	P
N5	Spouse	Woman	55	Senior middle school	Married	Liberal professions	Man	60	Primary school	Married	Agriculture	1	3	F	F	F
N6	Spouse	Man	63	An illiterate person	Married	Agriculture	Woman	66	Junior middle school	Married	Service/manual labor	2	3	F	F	P
N7	Son-in-law	Man	43	Undergraduate course	Married	Public functionary	Woman	68	An illiterate person	Married	Unemployed/other	3	3	F	F	P
N8	Brother and sister	Woman	47	Junior middle school	Divorced	Cleaning	Man	59	Junior middle school	Married	Service/manual labor	1	3	F	F	F
N9	Spouse	Man	58	Senior middle school	Married	Service/manual labor	Woman	64	An illiterate person	Married	Staff and workers	2	3	F	F	P
N10	Sons and daughters	Woman	35	Master	Unmarried	Healthcare/education	Man	67	Primary school	Bereft of one's spouse	Retired	1	3	F	F	F
N11	Spouse	Man	61	Junior middle school	Married	Retired	Woman	63	Special school	Married	Dancer	1	3	F	F	P
N12	Brother and sister	Woman	45	Undergraduate course	Married	Healthcare/education	Man	60	Junior middle school	Married	Service/manual labor	3	3	F	F	F
N13	Spouse	Woman	59	An illiterate person	Married	Unemployed/other	Man	69	Primary school	Married	Unemployed/other	2	3	F	F	F
N14	Sons and daughters	Man	29	Undergraduate course	Unmarried	Technical/professional	Woman	58	Senior middle school	Married	Staff and workers	1	3	F	F	P
N15	Father and daughter	Woman	36	Junior college	Married	Clerical staff	Man	74	An illiterate person	Married	Retired	2	3	F	F	F
N16	Mother and son	Man	40	Undergraduate course	Divorced	Constructor	Woman	62	Special school	Married	Staff and workers	1	3	F	F	P

T1 after admission, T2 before discharge, T3 one month after discharge. Interview form: F face to face, P telephone.

Damage grading: Grade 0 indicates a normal joint space without osteophytes or cartilage damage. Grade I refers to mild joint space narrowing with possible tiny osteophytes, usually accompanied by occasional mild pain that worsens after activity. Grade II is characterized by definite osteophyte formation and moderate joint space narrowing (less than 50%), with noticeable pain during stair climbing or after prolonged sitting. Grade III represents significant joint space narrowing (greater than 50%) and subchondral bone sclerosis, with daily activities becoming restricted and possible joint swelling. Grade IV describes complete joint space obliteration with extensive osteophyte formation and bone deformity, leading to persistent pain, stiffness, and difficulty or even inability to walk.

### 3.2 Theme 1: care tasks

Caregiving tasks expanded over time from logistical coordination to complex, quasi-clinical responsibilities. In the diagnosis stage, caregivers focused on obtaining medical information, organizing hospital admission, and negotiating treatment decisions. During discharge transition, tasks shifted toward facilitating functional training, modifying the home environment, and acquiring practical caregiving skills. In rehabilitation, responsibilities included preventing complications, managing long-term functional recovery, and sustaining patients’ psychological adaptation. Differences between subgroups emerged: spouses more often engaged in continuous physical assistance, whereas adult children were more likely to coordinate external resources and financial support. This transition illustrates a trajectory of increasing technical and psychosocial complexity (Table 2).

### 3.3 Theme 2: emotional experiences

Emotional states varied across phases, with acute anxiety and helplessness dominating the diagnosis stage. During discharge transition, caregivers reported fatigue, tension, and insecurity regarding recovery. By the rehabilitation phase, emotions became ambivalent, combining hope with frustration at slow progress, and in some cases, role-related self-sacrifice. Female caregivers more frequently expressed emotional exhaustion and feelings of isolation, whereas male caregivers emphasized financial pressure and role strain. This heterogeneity underscores how caregiver identity shapes psychological vulnerability and coping strategies (Table 4).

**Table 4:** Emotional themes of the primary caregivers of TKA patients at 3 periods.

Period	Theme	Sub-themes	Encoding	Representative statements
Preoperative	Psychological fluctuations	Anxiety and worry	Fear of intraoperative risk and poor postoperative recovery	N2: “I can't sleep at night because I heard that others don't recover well after this operation.”
	Character shock	A sudden sense of responsibility	Responsibility for sudden medical decisions has increased dramatically	N6: “I don't even know how to start with all this.”
	Feeling of helplessness	Lack of support	The sense of powerlessness due to lack of information and lack of system support	N1: “No one told me what to do next. I was very confused.”
Hospital stay	Emotional exhaustion	Both physically and mentally exhausted	Long-term care and anxiety coexist, resulting in low mood	N16: “Running up and down the hospital every day is tiring for both my body and mind.”
	sense of guilt	I feel powerless	The guilt of not being able to alleviate the patient's suffering	N10: “I feel useless when he hurts me but can't help him.”
	Struggle to adapt	Character maladjustment	Adaptation to the transition of caregiving status	N9: “It was really hard for me to accept the transition from family member to caregiver.”
Post-hospitalization	Aloneness	Lack of social support	Lack of outside understanding and help	N4: “It would be nice if everyone went out of the hospital, but it's harder for me to take care of myself at home.”
	Persistent anxiety	Uncertainty about rehabilitation	Worried about the postoperative recovery effect and future independence	N8: “The doctor said it depends on the situation, and I'm scared every day.”
	Emotional repression	Sacrifice oneself	Neglect your own emotions and needs	N13: “I only think about whether he is well or not every day, and I don't care whether I am tired or not.”

### 3.4 Theme 3: caregiving barriers

Three categories of barriers were identified: (1) knowledge barriers, such as limited understanding of perioperative information; (2) skill barriers, including difficulties with mobility support, wound care, and complication prevention; and (3) systemic barriers, encompassing financial stress, fragmented care coordination, and insufficient community resources. These barriers intensified in rehabilitation, when institutional support diminished while care demands persisted. The findings reflect the uniqueness of the post-TKA context in China, where the family remains the primary care provider in the absence of structured community-based rehabilitation pathways (Table 5).

**Table 5:** Themes of care barriers in three periods for the primary caregivers of TKA patients.

Period	Theme	Sub-themes	Encoding	Representative statements
Preoperative	Knowledge barriers	Lack of knowledge about surgery	Lack of understanding of TKA procedures, postoperative risks and nursing requirements	N1: “The doctor spoke too fast for me to remember how to prepare.”
	Communication barriers	Poor communication with medical staff	I am afraid that my questions will be annoying, so I dare not communicate actively	N3: “He gets a little impatient when I ask the doctor a few questions, so I dare not ask more.”
	Lack of external support	Limited use of social resources	They do not understand the policy, medical treatment process and nursing resources	N6: “I don't know how to report medical insurance or how to hire a nurse. No one says anything.”
Hospital stay	Skill barriers	Lack of nursing skills	Do not know how to assist patients to turn over, go to the toilet, rehabilitation training	N5: “He can't move, and I dare not move him for fear of hurting him.”
	Resource barriers	Room conditions are limited	The care space is small and inconvenient	N8: “I sleep on a chair at night and it's not convenient to take a shower.”
	Time conflict	It's hard to balance family and work	It's impossible to take care of the patient and work	N10: “The unit has to go, the hospital has to come, it's really tiring to run back and forth.”
Post-hospitalization	Care skills impairment	Lack of home rehabilitation guidance	They do not know about home exercise and medication	N7: “I don't know how to help him exercise. I'm afraid I'll make a mistake."
	Economic pressures	Rehabilitation is expensive	It is necessary to buy auxiliary appliances and nutritional products, which is costly	N4: “The wheelchair and rehabilitation equipment alone cost thousands.”
	Lack of support systems	Family support is inadequate	Other family members are less involved in care and responsibilities are concentrated	N13: “I'm the only one who can do it. The rest of the family can't help.”

### 3.5 Theme 4: support systems

Support sources shifted from hospital-based professionals (orthopedics, anesthesiology, inpatient nurses) during the diagnosis and hospitalization phases to rehabilitation therapists and discharge planners in the transition phase, and finally to community services, counselors, and family networks in the rehabilitation phase. However, this transition also revealed fragmentation between hospital and community care, leaving caregivers without continuous professional guidance (Table 1).

## 4 Discussion

### 4.1 Dynamic evolution of care tasks and role transformation

This study used longitudinal qualitative interviews to systematically depict how caregiving tasks for TKA patients evolved across the diagnosis, discharge transition, and rehabilitation stages. The findings confirm that caregiving is not static but develops in a staged and accumulative manner. Caregivers began as logistical coordinators—responsible for admission, paperwork, and information gathering—and then shifted toward performing quasi-clinical duties, such as monitoring rehabilitation training, preventing complications, and supporting psychological recovery. By the rehabilitation phase, their roles increasingly resembled those of paraprofessionals, combining physical, technical, and emotional responsibilities.

This trajectory can be interpreted through role adaptation theory, which posits that individuals gradually reconstruct their roles in response to prolonged exposure to stressors and expectations. The evidence from this study enriches existing cross-sectional findings (18, 19) by demonstrating how caregivers actively adapt their roles over time. In particular, spousal caregivers were more consistently engaged in hands-on tasks, whereas adult children often took on resource coordination and financial responsibilities, illustrating how family role structures influence caregiving dynamics.

### 4.2 Stage-based emotional fluctuations interpreted through stress–coping models

Caregivers’ emotional responses shifted significantly across the three phases, reflecting the appraisal and coping processes described in Lazarus and Folkman's stress–coping model. In the diagnosis stage, anxiety and helplessness reflected primary appraisal of uncertain events, while the discharge transition period was characterized by secondary appraisal under conditions of insufficient coping resources, leading to fatigue and diminished confidence. By the rehabilitation stage, caregivers reported ambivalent emotions: hope and optimism about patient recovery, but also frustration with slow progress, psychological suppression, and even self-sacrifice.

The gender differences observed add cultural nuance. Female caregivers frequently emphasized emotional exhaustion and loneliness, while male caregivers reported financial pressure and role strain. Such differences are consistent with gendered expectations in Chinese families, where women are often expected to engage in emotional labor and men in financial support. Compared with Western studies (20, 21), our participants reported more emotional suppression and acceptance of sacrifice, suggesting the influence of filial piety and spousal duty in shaping emotional coping strategies in the Chinese context.

### 4.3 Theoretical and analytical considerations: framing caregiver burden

Beyond descriptive findings, the analysis benefits from a theoretical integration of the caregiver burden framework. This framework conceptualizes stress across informational, physical, and systemic domains, and our data highlight how these burdens accumulate longitudinally. Early deficits in medical information limited caregivers’ ability to make informed decisions. Skill-related challenges in patient transfer and complication prevention became most salient during discharge and early rehabilitation, when institutional guidance diminished. Systemic barriers—financial strain, lack of community rehabilitation services, and fragmented continuity of care—were most pronounced during long-term recovery.

These findings extend prior work (22, 23) by showing that caregiver burden is not simply a static condition but an escalating process tied to the temporal trajectory of recovery. Importantly, the theoretical framing underscores the need for stage-specific interventions. Structured preoperative education could address informational gaps, while community-based rehabilitation services and caregiver training programs could alleviate long-term skill deficits.

### 4.4 Evolution of support networks and cultural specificity

Support networks shifted from professional, hospital-based providers during admission and hospitalization to family and community resources during rehabilitation. This transition revealed fragmentation in the healthcare–community–home continuum, with caregivers often left unsupported after discharge. While similar patterns of fragmentation have been described internationally (24), the findings in this study reflect the particular reliance on family caregiving in China, where community rehabilitation and formal long-term care systems remain underdeveloped.

This cultural specificity is crucial: in China, caregiving remains deeply embedded in family structures, guided by norms of filial piety and collective responsibility. Unlike in some Western contexts where professional caregivers supplement family roles, Chinese families are often the sole source of support. This reliance underscores the importance of building multidisciplinary and community-integrated support systems that can bridge hospital discharge with ongoing rehabilitation.

### 4.5 The novelty of this study

An important contribution of this study lies in its methodological design. Previous research on TKA caregiving has been largely cross-sectional, providing valuable but static insights into caregiver burden, emotional distress, or support needs. By adopting a longitudinal design, this study captured temporal trajectories of caregiving tasks, emotional states, and barriers, revealing not only what caregivers experience but also when these challenges emerge and how they evolve.

For example, anxiety peaked during the diagnosis and discharge phases, while frustration and fatigue accumulated during rehabilitation. Similarly, systemic barriers were less visible during hospitalization but became acute once institutional support receded. Such insights are possible only with longitudinal observation, highlighting the added value of this approach for developing time-sensitive interventions.

### 4.6 Study limitations and future directions

This study has several limitations. First, participants were recruited from a single hospital, limiting generalizability to other regions. Second, although purposive sampling with maximum variation was employed, selection bias cannot be excluded; caregivers under extreme burden may have declined participation. Third, data were self-reported, and despite triangulation with non-verbal observations, recall and desirability bias may still have influenced findings. Fourth, the positionality of the researchers as clinical nurses may have affected interview dynamics, despite the use of reflexive journaling and peer debriefing to mitigate this risk. Fifth, the interview duration was relatively short (15–20 min) due to time constraints in clinical environments, which may have limited narrative depth.

Future studies should expand to multiple sites and diverse cultural settings, adopt mixed-methods approaches to triangulate qualitative findings with quantitative measures of caregiver burden, and extend follow-up periods to capture long-term trajectories beyond the first month of rehabilitation.

## 5 Conclusion

This longitudinal qualitative study mapped the evolving care journey of primary caregivers of TKA patients across the diagnosis, discharge transition, and rehabilitation stages. The findings revealed that caregivers encountered cumulative burdens, emotional fluctuations, knowledge and skill gaps, and insufficient systemic support at each stage, with their roles continuously shifting and intensifying. These insights underscore the need for stage-specific interventions. In the preoperative phase, structured caregiver education programs should be established to provide accurate medical knowledge, set realistic expectations, and prepare caregivers for their roles. During the discharge transition, multidisciplinary discharge planning combined with short-term psychological counseling is critical to reduce anxiety, enhance confidence, and ensure effective continuity of care. In the rehabilitation stage, caregivers would benefit most from community-based rehabilitation services, peer-support groups, and digital training platforms, which can sustain skill development, alleviate long-term burden, and prevent social isolation. Overall, a caregiver-centered, culturally sensitive, and multidisciplinary continuum of care should be prioritized to strengthen caregiving quality and mitigate both the physical and emotional strain on TKA caregivers.

## Data Availability

The raw data supporting the conclusions of this article will be made available by the authors, without undue reservation.
